# Olanzapine-Induced Black Hairy Tongue During Treatment of Hebephrenic Schizophrenia Decompensation: A Rare Case Report and Brief Review of the Literature

**DOI:** 10.7759/cureus.75044

**Published:** 2024-12-03

**Authors:** Rita Ortiga, Maria B Couto, Elisa Lopes, João B Fonseca

**Affiliations:** 1 Department of Psychiatry, Unidade Local de Saúde do Alto Ave, Guimarães, PRT

**Keywords:** adverse drug reactions, black hairy tongue, case report, hebephrenic schizophrenia, olanzapine, schizophrenia

## Abstract

Black hairy tongue (BHT) is a benign, self-limiting, and usually asymptomatic condition, characterized by abnormally hypertrophied and elongated filiform papillae on the surface of the tongue. In this article, we present the case of a woman diagnosed with hebephrenic schizophrenia who developed BHT after using olanzapine to treat an acute episode of the disease. The temporal coincidence between the development of BHT and the increase in olanzapine dosage to 20 mg daily suggests a likely dose-dependent relationship, making this psychotropic drug the most probable cause of this condition. Although its etiology remains undefined, several factors have been suggested, namely antipsychotics and cannabinoids, likely due to the xerostomia they induce. In this patient, BHT was successfully treated by improving oral hygiene and discontinuing the use of olanzapine and cannabinoids.

## Introduction

Black hairy tongue (BHT) is a benign, self-limiting condition characterized by abnormally hypertrophied and elongated filiform papillae on the surface of the tongue, originally described by Amatus Lusitanus in 1557 [1-3]. There are numerous synonyms for BHT, including hyperkeratosis of the tongue, lingua villosa nigra, *nigrities linguae*, kerato-mycosis lingual, lingua villosa, and melanotrichia lingual [3]. In addition, the hairy tongue may appear in colors other than black, such as brown, yellow, green, blue, or even unpigmented [1-3]. Its prevalence varies widely between studies, ranging from 0.0% to 53.8% [1-3].

Typically, the main reason for seeking medical attention is aesthetic concerns, as it is generally an asymptomatic condition [1,2]. However, in rare cases, BHT may cause choking, nausea, dysgeusia, a metallic taste, xerostomia, burning mouth syndrome, tickling, halitosis, and even cervical or submandibular lymphadenopathy [1-3].

BHT is thought to result from defective desquamation of the dorsal surface of the tongue [1-3]. This prevents normal debridement and leads to the accumulation of keratinized layers, resulting in excessive growth and thickening of the filiform papillae, which then collect bacteria, fungi, or other debris [1-3]. This accumulated foreign material usually contributes to discoloration [1-3]. However, the etiology and pathophysiology are not fully understood and are likely multifactorial [1,2].

The differential diagnosis for BHT includes pseudo-BHT (it resembles a tongue stained darkly in the absence of elongated filiform papillae, caused by chemicals or food coloring), oral hairy leukoplakia, pigmented fungiform papillae of the tongue, acanthosis nigricans, congenital lingual melanotic macules, congenital melanocytic nevi, premalignant leukoplakia, squamous cell carcinoma, and hypertrophic *Herpes Simplex* virus infection [1,2].

There are no objective diagnostic criteria for this condition [1]. The diagnosis of BHT relies primarily on a thorough history and intraoral visual examination [1-3]. BHT has a predilection for the dorsal aspect of the tongue, anterior to the circumvallate papillae, and the terminal sulcus [1-3]. It generally does not occur on the tip or sides of the tongue [1,2]. Microscopic examination may be used to aid in diagnosis [2,3]. The elongated papillae are usually less than 1 mm in length, but may reach lengths of 12-18 mm and a width of 2 mm [1,2]. Cultures may be considered to rule out superimposed bacterial or fungal infections associated with BHT [2]. Although a tongue biopsy is generally not considered necessary, if the lesion is atypical, refractory to treatment, or symptomatic, the clinician should consider a biopsy as the suspicion for malignancy or systemic disease should be high [1,2].

A careful review of known precipitating factors and recent medication changes is also fundamental to the diagnosis of BHT [1-3]. Several drugs have been associated with the development of BHT, such as amoxicillin/clavulanic acid, aureomycin, benztropine mesylate, chlorpromazine, clonazepam, doxycycline, erlotinib, erythromycin, fluoxetine, lansoprazole, linezolid, lithium, mepazine, methyldopa, neomycin, olanzapine, paroxetine, PEG-interferon, penicillin, tetracycline, and thiothixene hydrochloride [1-3]. Other non-medical factors also associated with an increased risk of developing BHT include alcohol, drugs of abuse (particularly intravenous or smoking drugs such as crack cocaine), conditions of edentulous patients, the elderly, excessive coffee consumption, heavy black tea consumption, male gender, oxidizing mouthwashes (containing sodium perborate, sodium peroxide, and hydrogen peroxide), poor oral hygiene, soft or pureed diets, and tobacco (smoking, chewing) [1-3]. Medical conditions such as cancer, chronic dry mouth, immunocompromised states (acquired immunodeficiency syndrome, graft-versus-host disease, and others), amyotrophic lateral sclerosis, recent radiation therapy (particularly to the head and neck region), and trigeminal neuralgia have also been shown to be related to BHT [1-3].

Schizophrenia affects about 1% of the world's population, usually begins in the first decades of life, and often has a chronic course with significant deterioration [4]. Although it has fallen into disuse, schizophrenia was once classified into five subtypes based mainly on clinical presentation: paranoid, hebephrenic or disorganized, catatonic, undifferentiated, and residual [5,6]. The hebephrenic subtype is included in this article because the authors believe it may be relevant for a better understanding of the clinical case that follows. This subtype appears early in life, usually before the age of 25, and is characterized by prominent affective symptoms and changes in behavior and thinking [5,6]. In this subtype, there is a marked regression to primitive, disinhibited, and disorganized behavior, but without symptoms that meet the criteria for the catatonic subtype [5,6]. The appearance is sloppy, and the mood is erratic, with inappropriate emotional responses, often bursting into laughter for no apparent reason [5,6]. Incongruous smiles and grimaces are also common in these patients, whose behavior is best described as erratic or unpredictable [5,6]. Mannerisms are common [5,6]. Delusions and hallucinations are episodic and unsystematic [5,6]. Speech is disjointed and incoherent, reflecting thought disorder [5,6]. Negative symptoms appear early and contribute to a poor prognosis [6].

Antipsychotics are the mainstay of treatment for schizophrenia [6]. There are no significant differences in efficacy between different antipsychotics, except for clozapine [4,6]. However, this does not mean that all antipsychotics are the same, as there are considerable differences in their side-effect profiles [4,6]. Furthermore, atypical antipsychotics (or second-generation antipsychotics) are currently considered first-line medications for treating schizophrenia according to major clinical guidelines [4]. In general, the choice of medication should be based on the patient's preference, the effects of previous treatments, and the relative likelihood of the medication causing serious side effects [6].

Olanzapine is commonly used in the treatment of schizophrenia in patients 12 years of age or older [7]. It is also approved for maintaining response in the long-term treatment of schizophrenia and for episodes of agitation associated with it [7]. The minimum effective dose is 5 mg daily for the first episode of psychosis in schizophrenia, and 10 mg daily for relapses [7]. The maximum recommended dose is 20 mg daily [7]. Olanzapine acts as an antagonist of dopamine (D2) and serotonin (5HT2A and 5HT2C) receptors, although it also affects other neurotransmitter receptors [7]. The primary symptoms targeted by olanzapine include the positive, negative, and cognitive symptoms of psychosis, in addition to mood instability and aggressive symptoms [7]. The most notable adverse effects of olanzapine are increased appetite, weight gain, dizziness, sedation, dry mouth, constipation, dyspepsia, arthralgias, edema, and increased risk of diabetes mellitus and dyslipidemia [7].

In this article, we present the case of a woman diagnosed with hebephrenic schizophrenia who developed BHT after using olanzapine to treat an acute episode of the disease.

## Case presentation

A 56-year-old woman diagnosed with hebephrenic schizophrenia has a history of multiple hospitalizations for consistent symptom patterns characterized by marked behavioral, thought, and mood disorganization, all requiring electroconvulsive therapy (ECT) for stabilization. She has never been offered maintenance ECT (mECT). Her first psychotic break occurred at the end of her final year in a math and science degree program, and she has never worked since. The patient has no other relevant medical history, but her father had bipolar affective disorder.

She was involuntarily hospitalized for psychotic decompensation, possibly triggered by her father's death and recent cannabinoid abuse. Cannabinoid use had started approximately three months prior, and there was no use of other psychoactive substances, including alcohol and tobacco. Non-adherence to pharmacological therapy was not noted.

The patient's condition progressively worsened over the course of three weeks, a trend that continued throughout her hospitalization. She exhibited constant and unpredictable behavioral fluctuations, alternating between postures demonstrating fear with heteroaggression and a childlike, overly familiar demeanor. Containment measures, including physical, chemical, and environmental interventions, were frequently required. Initially, she presented with infantilized, verbose, and strident speech, full of neologisms and paralogisms that evolved into word salad. She displayed parathymia, alternating between crying, laughing, and screaming in the same observation. Formal thought disorders were the most prominent, culminating in driveling. She also presented paranoid delusions with reduced systematization, delusional pregnancy (the baby would be a Pikachu), Fregoli delusion, and visual, auditory, and cenesthetic hallucinations.

The patient underwent extensive analytical and imaging studies on admission. The analytical study revealed a pattern of hepatic cytolysis (liver enzymes at levels approximately five times the upper limit of normal), with no other significant changes, including blood counts, ionogram, renal function, thyroid hormones, vitamins and minerals, inflammatory parameters, viral serologies, and syphilis screening. Urine drug screens were positive for cannabinoids. Serum valproic acid levels were within the therapeutic range. Cranioencephalic computed tomography and electrocardiogram showed no relevant changes. Physical examination and vital signs were within normal limits.

The attending outpatient psychiatrist clarified that the pattern of hepatic cytolysis had been persistent over the past few years. Consultation was sought with the internal medicine department, who suggested an abdominal ultrasound. This revealed a pattern of moderate diffuse hepatic steatosis with no other relevant findings. The internists related the analytical and imaging findings to drug iatrogenesis and recommended dietary measures, physical exercise, and, if possible, therapeutic review.

Given the exuberance of the clinical presentation, several therapeutic regimens were tried. In the outpatient clinic, the patient was treated with valproic acid at a dose of 500 mg daily, paliperidone 6 mg daily, quetiapine 100 mg daily, and lorazepam 7.5 mg daily. On admission, the doses of quetiapine and paliperidone were increased to 200 mg daily and 9 mg daily, respectively, and oxazepam 100 mg daily was started.

On the third day of hospitalization, the patient's clinical condition continued to deteriorate, presenting severe psychomotor agitation associated with heteroaggressiveness, so quetiapine was suspended and olanzapine 10 mg daily and trazodone 100 mg daily were started, the latter increased to a dose of 200 mg daily, but without any response.

The state of psychomotor agitation was so severe that it caused rhabdomyolysis with an increase in creatine kinase enzyme levels to approximately 13 times the upper limit of normal. Therefore, on the fifth day of hospitalization, oxazepam was replaced by clonazepam 4 mg daily and the dose of olanzapine was increased to 20 mg daily.

**Figure 1:**
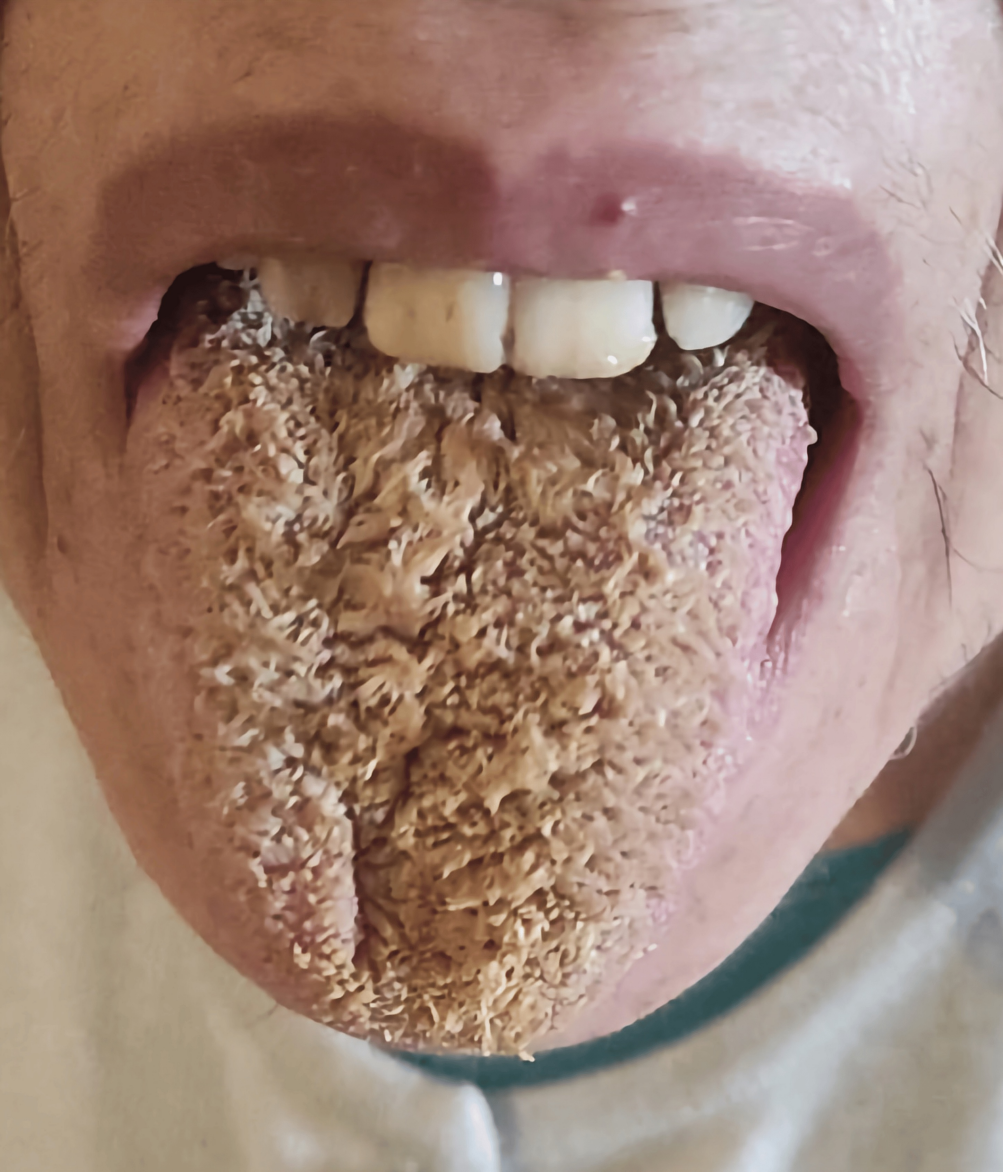
Black hairy tongue on the first day of weaning from olanzapine. Consent for publishing the image was obtained from the patient.

On the ninth day of hospitalization, four days after increasing the dose of olanzapine to 20 mg daily, a light brown color was observed on the patient's tongue. With the clinical suspicion of oral candidiasis, nystatin 1 ml daily was started. However, two days later, the exuberance of the patient's tongue called this diagnosis into question. Examination of the oral cavity revealed a tongue with light brown hair-like projections covering the dorsal surface but sparing the lateral regions (Figure 1). No other changes were observed in the rest of the oral cavity except for evidence of poor oral hygiene. The patient denied any pain or discomfort in the tongue. There was no recent use of alcohol, tobacco, or excessively pigmented or sugary drinks and foods. It is important to clarify that in the initial phase of the hospitalization, the provision of oral hygiene care supported by the nursing team was clearly hampered by the patient's permanent state of psychomotor agitation. At this point, a brief literature search was performed, and the diagnosis of olanzapine-induced BHT was considered. Therefore, progressive weaning of olanzapine began that same day, and consultancy was requested by the stomatology specialty. The stomatologists agreed with the diagnosis and the probable association with olanzapine. In addition, they suggested stopping nystatin, since the hypothesis of oral candidiasis was ruled out, and they waived the need for additional diagnostic tests. They also reinforced the improvement of oral hygiene care, recommending brushing teeth and tongue three times a day with a soft toothbrush that should be changed monthly. They also promoted the reinforcement of oral hydration, the avoidance of consuming foods or drinks that are too pigmented and sugary, alcoholic beverages, and tobacco or other smoking substances.

**Figure 2:**
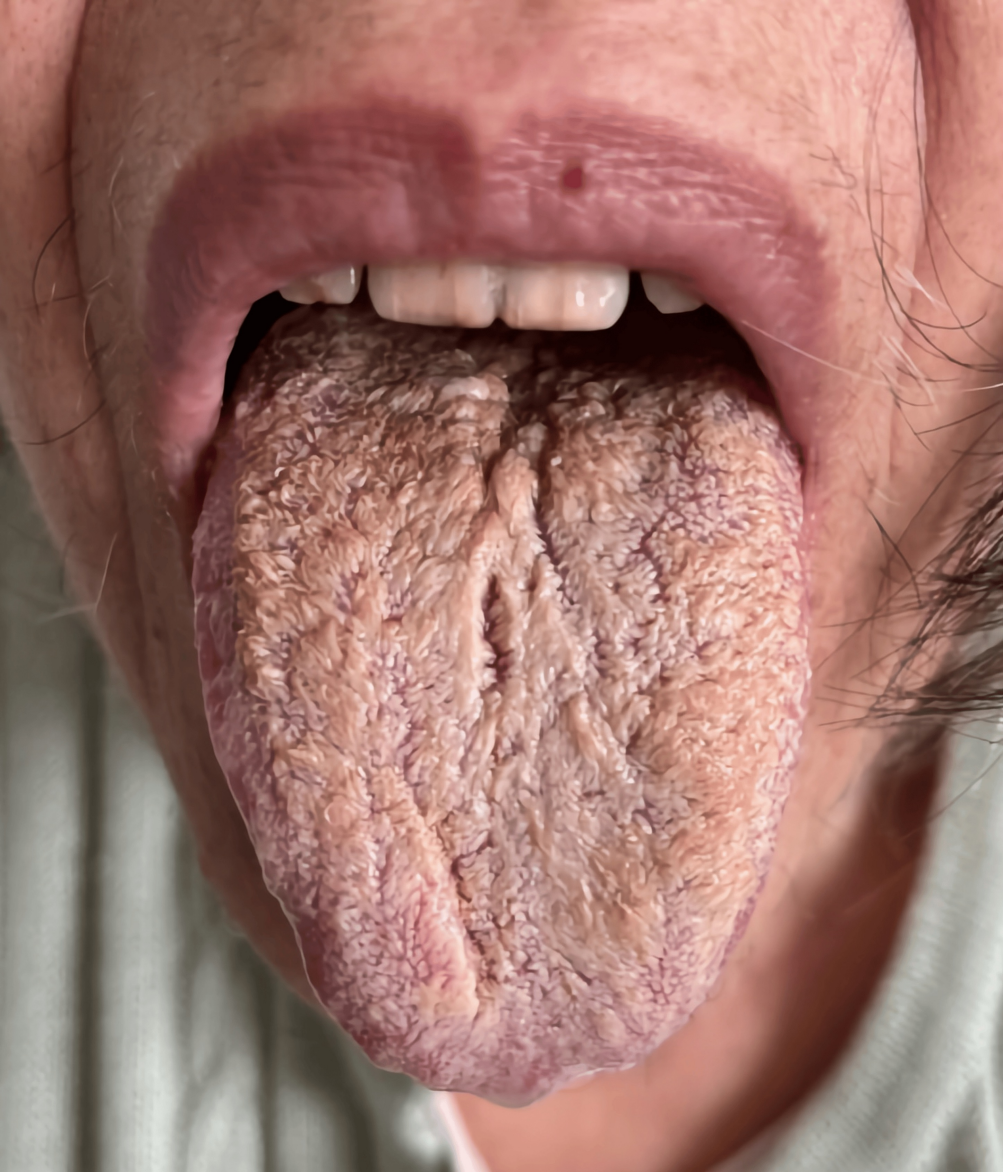
Black hairy tongue on the 15th day of weaning from olanzapine. Consent for publishing the image was obtained from the patient.

**Figure 3:**
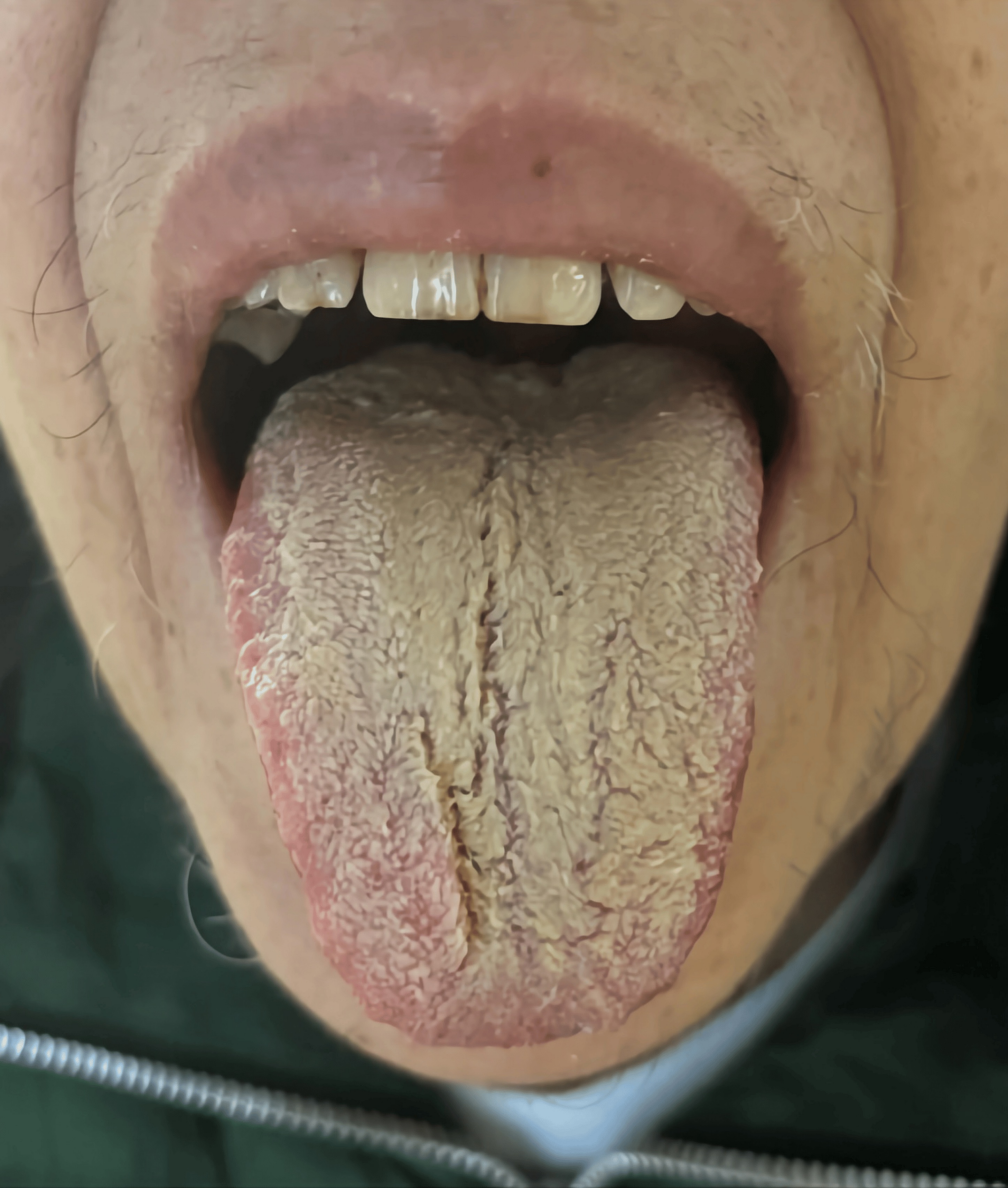
Black hairy tongue on the day of complete discontinuation of olanzapine. Consent to publish the image was obtained from the patient.

**Figure 4:**
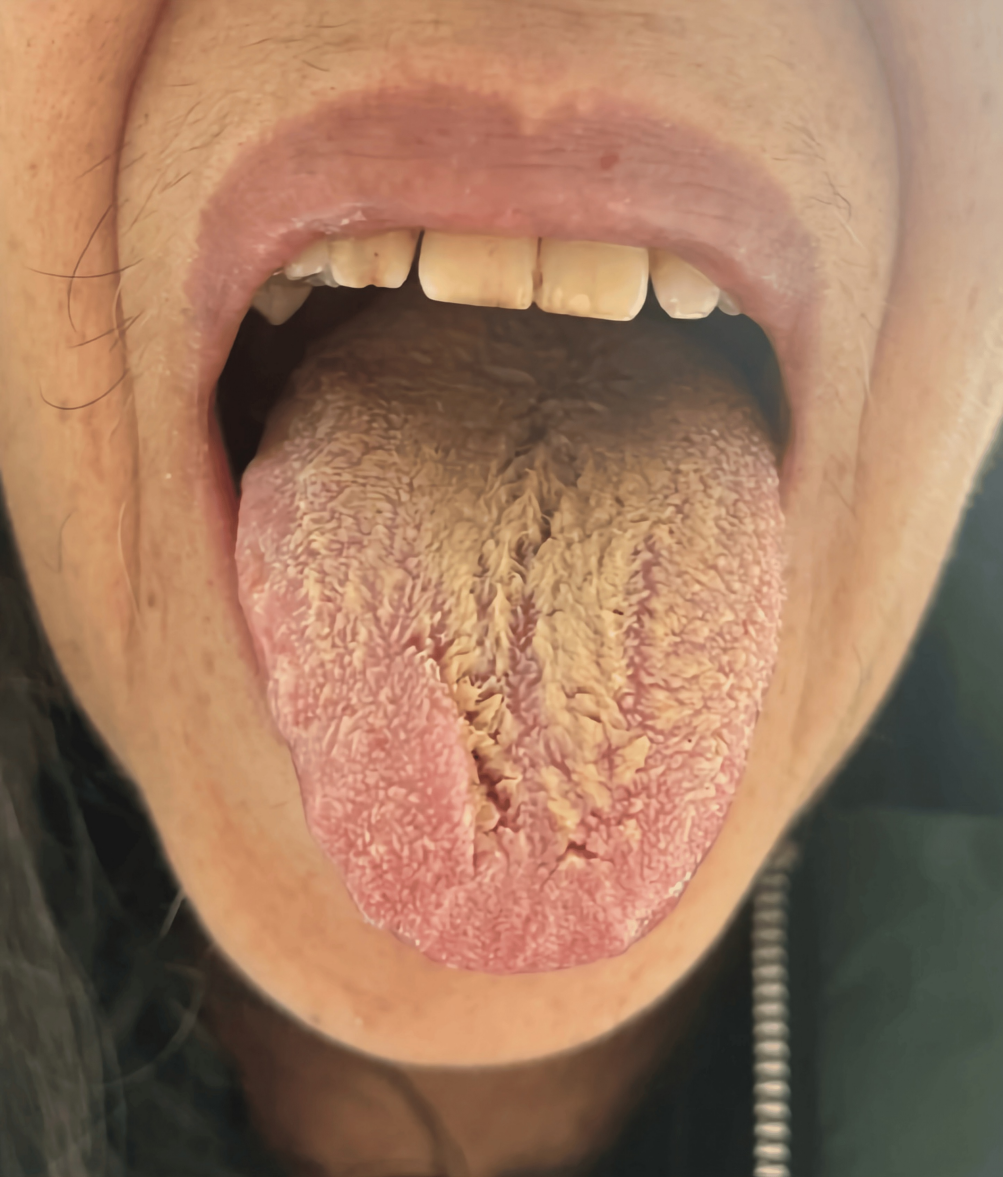
Black hairy tongue five days after complete discontinuation of olanzapine. Consent for publishing the image was obtained from the patient.

**Figure 5:**
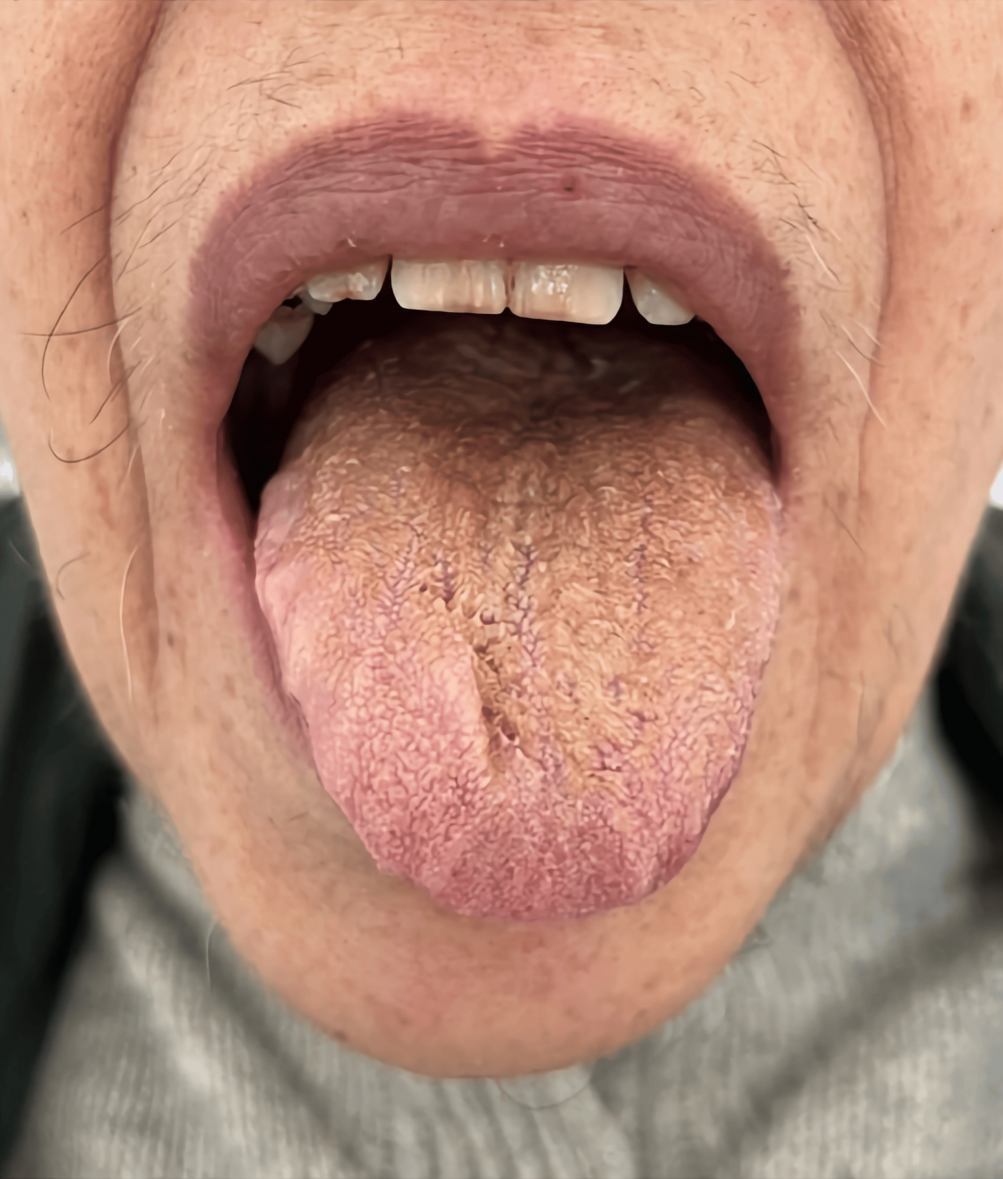
Black hairy tongue 10 days after discontinuation of olanzapine. Consent to publish the image was obtained from the patient.

As the patient maintained severe behavioral changes and began to taper off olanzapine, chlorpromazine 50 mg daily was introduced due to its sedating profile and subsequently increased to a dose of 100 mg daily. Despite the introduction of this new medication, BHT did not worsen and began to improve a few days after weaning from olanzapine. Olanzapine was finally discontinued on the 39th day of hospitalization, 28 days after the start of weaning. The sequence of Figures 2-5 shows the favorable evolution of BHT from its onset to the date of hospital discharge.

Unfortunately, psychopathological changes did not show the same favorable evolution, and drug iatrogenesis proved to be significant. Overall, in addition to BHT, the most significant adverse effects were hepatic cytolysis, rhabdomyolysis, and severe constipation. For all these reasons, it was decided to start ECT on the 17th day of hospitalization. The patient underwent a total of 12 ECT sessions, carried out three times a week, without any record of relevant complications. Psychopathological improvement was noted after the third session, which allowed the dose of other medications to be reduced, including paliperidone and chlorpromazine. At the end of treatment, the patient showed stabilized behavior, coherent thinking and speech, improved mood, and no psychotic symptoms.

**Figure 6:**
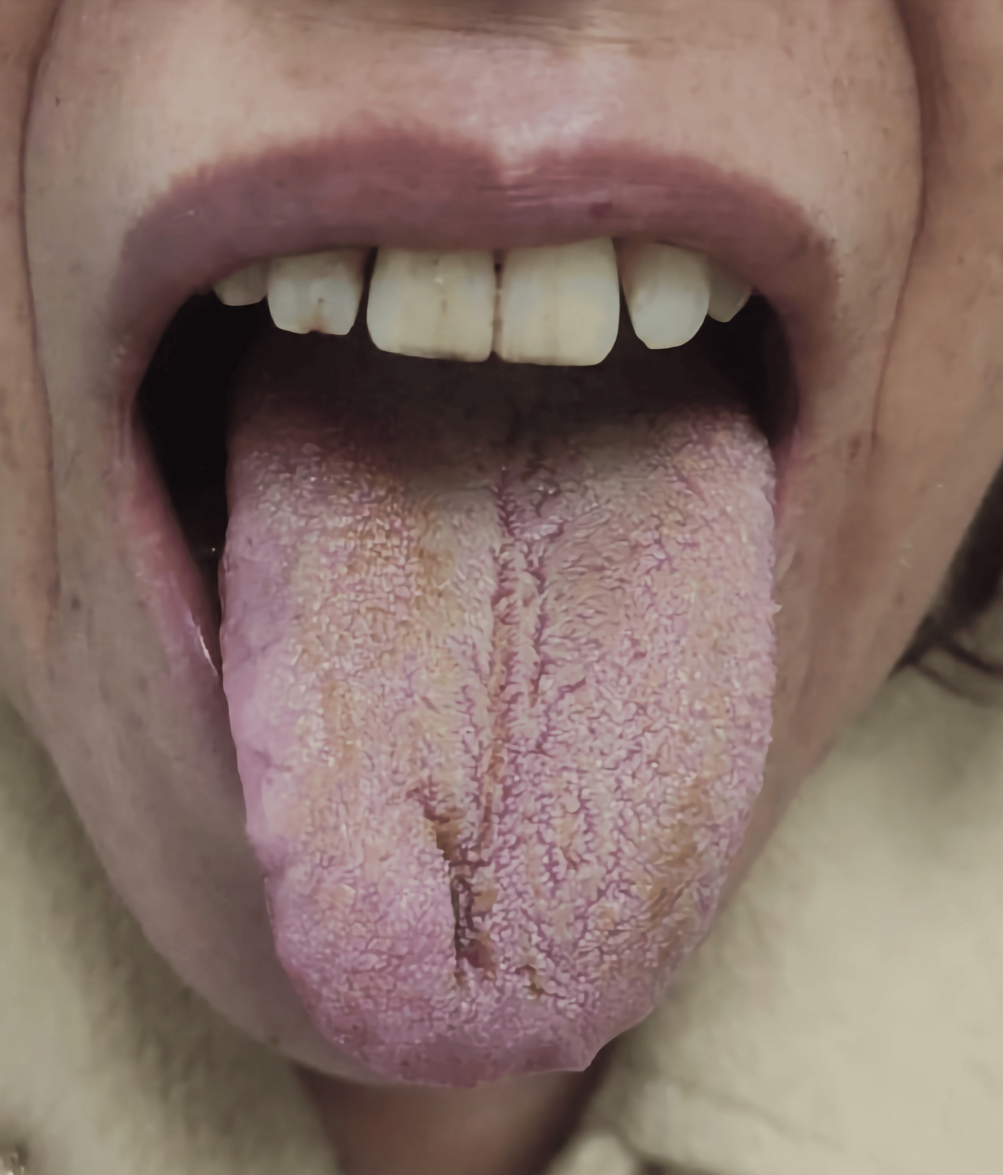
Black hairy tongue two months after discharge from the hospital. Consent to publish the image was obtained from the patient.

The patient was discharged after 50 days of hospitalization, medicated with valproic acid 500 mg daily, paliperidone 6 mg daily, chlorpromazine 75 mg daily, trazodone 200 mg daily, and clonazepam 4 mg daily. Monthly mECT was suggested at discharge, which the patient agreed to. Currently, she is psychopathologically stabilized in an outpatient clinic, and the BHT is in almost complete remission. The remaining complications caused by drug iatrogenesis have also resolved. Figure 6 shows the patient's tongue two months after discharge from the hospital.

## Discussion

The temporal coincidence between the development of BHT and the increase in olanzapine dose to 20 mg daily makes this psychotropic drug the most likely cause of the injury. This is supported by a Naranjo Adverse Drug Reaction Probability Scale score of five, which indicates that the reaction is likely caused by olanzapine [3].

However, the patient has other factors in her clinical history that are considered risk factors for the development of BHT. Among these factors is poor oral hygiene, partly conditioned by hebephrenic schizophrenia itself, which often leads to a lack of self-care in the context of the typical pattern of severe behavioral disorganization, especially in phases of decompensation such as the one in which the patient found herself [1-3,5]. This also conditioned the provision of oral hygiene care by third parties, both in the prehospital phase and during hospitalization. However, despite a long history of psychotic decompensation episodes of similar severity, there was no record of the development of this condition. Furthermore, BHT did not occur until the ninth day of hospitalization, further supporting the relationship with the introduction of olanzapine four days earlier.

There are case reports of BHT induced by both chlorpromazine and clonazepam, drugs introduced during hospitalization that can cause xerostomia, a risk factor for BHT [7]. Chlorpromazine was introduced after the onset of BHT to compensate for the weaning of olanzapine, as the patient continued to have severe psychopathology. Furthermore, the dose was increased at this stage, and there was no worsening or stagnation of BHT. Clonazepam was introduced on the same day that olanzapine was increased to 20 mg daily, an important milestone in the clinical course. Nevertheless, the lesion began to diminish after weaning from olanzapine, and the patient continues to be treated with the same dose of clonazepam without recurrence of the lesion, also making the involvement of this medication unlikely. Although we highlight these medications, it is true that most medications taken by the patient both on admission and during hospitalization have the potential to cause xerostomia, although we have not found case reports directly linking them to the development of BHT [1-3,7]. For example, trazodone was introduced on admission and its dose was increased before and after the appearance of BHT without having any influence on the favorable evolution of the lesion [7]. The dose of quetiapine was also increased before the appearance of the lesion, and the medication was discontinued when olanzapine was introduced, without any signs suggesting BHT having been detected before [7]. As for paliperidone and valproic acid, in addition to the lack of case reports linking them to BHT, xerostomia is an unlikely adverse effect associated with them and the patient was taking these medications before admission and continues to take them on an outpatient basis, with no recurrence of the oral pathology [7].

Cannabis use is associated with dry mouth and hyposalivation via a CB_1_/CB_2_ receptor-mediated delta-9-tetrahydrocannabinol (THC) effect on salivary gland cholinergic transmission [8]. Dry mouth associated with cannabis use is reported to be similar to that associated with cigarette smoking, and most subjects experience dry mouth immediately after cannabis use [8]. Since xerostomia predisposes to the development of BHT, the use of smoked cannabis may have contributed to the development of this reaction [1-3,8,9]. However, the patient had started taking cannabinoids three months prior without developing any injury before admission, so olanzapine still seems to be the most robust hypothesis. Nevertheless, cessation of cannabinoid use is still strongly recommended for this patient, both to prevent oral pathology and to contribute to the management of schizophrenia [5,6,8].

In this patient, the relationship between olanzapine and BHT may have been dose-dependent, as sustained administration of olanzapine at a moderately lower dose (5-10 mg daily) did not cause progression or persistence of the lesion. The 20 mg dosage appears to be critical in this regard, as other case reports of olanzapine-induced BHT have also been noted when titrated to this dosage [10-12]. The most likely pathophysiological mechanism behind olanzapine-induced BHT appears to be this drug’s muscarinic receptor (M1) antagonism, causing anticholinergic effects such as xerostomia, which may contribute to defective desquamation of the dorsal region of the tongue [1-3,7].

Finally, a note regarding ECT: Unfortunately, ECT is often stigmatized and underutilized, especially mECT, leading to its discontinuation once the clinical goal is achieved [13]. We hypothesize that earlier referral to mECT could have prevented disease progression and multiple hospitalizations with resistance and intolerance to psychotropic medications, as it proved to be an effective therapy for this patient's acute episodes [13].

## Conclusions

The temporal coincidence between the development of BHT and the increase in olanzapine dosage to 20 mg daily made this psychotropic drug the most likely cause of this injury, suggesting a dose-dependent relationship. Any agents with a possible side effect of xerostomia can be a source of BHT, especially when administered in combination. In this patient, BHT was successfully treated by improving oral hygiene and discontinuing olanzapine and cannabinoid consumption. Clinicians should be aware of the prevalence, predisposing factors, and drug classes that may play a role in the development and treatment of BHT.
